# Corporate Application of Health Literacy

**DOI:** 10.3928/24748307-20210710-01

**Published:** 2021-07

**Authors:** Natascha Hochmuth, Kristine Sørensen

## Abstract

**Background:**

The growing concern of low health literacy in populations combined with the interest of companies to develop cultures of health and the emerging interest in the workplace as an arena for improving health literacy is increasingly acknowledged. This study aimed to explore how companies currently apply the concept of health literacy as part of their health efforts in the work sphere.

**Methods:**

A systematic literature review was conducted based on the PRISMA (Preferred Reporting Items for Systematic Reviews and Meta-analysis) guidelines. The search was performed in the databases PubMed, Science Direct, and Directory of Open Access Journals from July 2019 to August 2019 and included the search terms “health literacy,” “health literate,” and “health competence” combined with the search terms “company/companies,” “corporation/corporates,” “workplace,” “business,” and “organization.” Studies were eligible for inclusion if they (1) were written in English or German, (2) were concerned with employers/employees, companies, corporations, or organizations, (3) dealt with health literacy in particular, and (4) were formatted as journal articles, systematic literature reviews, or book chapters.

**Key Results:**

The search identified 20 articles. A thematic analysis resulted in 5 clusters with 2 to 3 sub-themes illustrating the characteristics of how corporations apply health literacy. The clusters entailed the conceptualization of health literacy, its measurement, and the application of health literacy in strategies, interventions, and training.

**Discussion:**

The literature review reveals that the application of health literacy by companies is in its infancy. More research and practical experience are warranted on how companies can mobilize and facilitate a health literate workforce. **[*HLRP: Health Literacy Research and Practice*. 2021;5(3):e218–e225.]**

**Plain Language Summary:**

This literature study explored the role of advancing health literacy in companies. Five aspects were identified as important, including the understanding of the concept, its measurement, and its integration into strategies, interventions, and training. The involvement of management and the staff was crucial for success.

Companies choosing health and productivity as a business strategy are on the rise (Loeppke et al., 2009). Keeping the workforce healthy and safe has proven to be a competitive business advantage (Gunther et al., 2019). Also, the integration of health literacy as an asset for corporate social responsibility and corporate health has been indicated (Sørensen & Brand, 2011). Due to mounting public pressure and rising consumer expectations, enterprises are increasingly expected to go beyond their legal requirements and act more responsibly (World Health Organization, 2011). Hence, many companies have promoted health by embracing “a triple bottom line” entailing “people, planet, and profits,” thereby also embracing the “corporate determinants of health” (Millar, 2013). A competitive marketplace requires the employers to take a holistic approach to access and mitigating health risks in their workforce, because employers with higher corporate health assessment scores, a common way to quantify “cultures of health,” tend to have a lower health care cost trend, without the need to reduce benefit services or shift more costs to their employees (Gunther et al., 2019). A “culture of health” is defined as one in which people and social entities (e.g., households, organizations) make healthy life choices within a broader social environment that values, provides, and promotes options that can produce health and well-being for everyone regardless of background or environment (Robert Wood Johnson Foundation, 2019). In terms of corporations, the engagement in employee, environmental, consumer, and community health is collectively referred to as the “corporate culture of health” (Kyle et al., 2019).

An investment in health literacy by companies is beneficial as research reveals limited health literacy to be a neglected public health challenge. The European Health Literacy Survey, for example, indicated that more than one-third of populations in eight countries in Europe might face difficulties in managing health and navigating health services (Sørensen, Pelikan, et al., 2015). Health literacy can be defined as a “people's knowledge, motivation, and competences to access, understand, appraise, and apply health information to make judgments and take decisions in everyday life concerning health care, disease prevention, and health promotion to maintain or improve quality of life during the life course” (Sørensen et al., 2012). Politically, the improvement of health literacy at work has gained traction (Kickbusch, 2012). Employers and corporate organizations can play an active role in helping their staff acquire the skills needed to manage their health as well as the health of their families and communities (World Health Organization, 2013). For example, the National Action Plan to Improve Health Literacy in the United States has specific goals and strategies for employers. They include (1) the development of workplace policies that increase and improve health information and services for employees and their families, (2) the assurance that information and services are culturally and linguistically appropriate, (3) the engagement of employees in evaluating health and wellness information, (4) when selecting existing health and insurance information products, they choose products that have been developed using health literacy principles and are culturally and linguistically appropriate, (5) consultation with local librarians to build an appropriate collection of health and insurance information products and connect with community resources, (6) negotiation with health insurers to provide employee-tested health information and the assurance that the information is culturally and linguistically appropriate, and (7) the provision of training, tools, and resources for employees to improve their health information-seeking and decision-making skills (U.S. Department of Health and Human Services. Office of Disease Prevention and Health Promotion, 2010). Moreover, employers can adopt ten attributes to develop health literate organizations (Brach, 2017; Brach, Keller, et al., 2012). Consequently, building health literate workplaces becomes a call to action for the employers, not just for each worker (Wong et al., 2012).

The specific type of health literacy related to health at work is often called “occupational health literacy.” Occupational health literacy is the degree to which workers have the capacity to obtain, communicate, process, and understand occupational health and safety information and services to make appropriate health decisions in the workplace (Rauscher & Myers, 2014). Rauscher and Myers (2014) found that occupational health literacy is positively associated with work-related injury prevalence and the receipt of safety training. Recent health literacy models pursue an even more integrated approach, focusing on risks as well as assets to understand fully health behavior in the workplace. These models address individual, organizational, and interpersonal factors concomitantly (Larsen et al., 2015). This approach is in line with Nutbeam (2008), who highlighted that health literacy should be considered not only a risk, but also an asset for people, organizations, communities, and societies.

In summary, recognizing the growing concern of low health literacy in populations combined with the interest of corporations to develop cultures of health and the emerging interest in the workplace as an arena for improving health literacy, this study aimed to explore how companies currently apply the concept of health literacy as part of their health efforts in the work sphere.

## Methods

The study design entailed a systematic literature review based on the PRISMA (Preferred Reporting Items for Systematic Reviews and Meta-analysis) guidelines (Moher et al., 2015). The search strategy included the search terms “health literacy,” “health literate,” and “health competence,” which were combined with the search terms “company/companies,” “corporation/corporate,” “workplace,” “business,” and “organization.” Studies were eligible for inclusion if they (1) were written in English or German, (2) were concerned with employers/employees, companies, or organizations, (3) dealt with health literacy in particular, and (4) were formatted as journal articles, systematic literature reviews, or book chapters. Studies were excluded if they were, for example, concerned with health literacy in children or adolescents, dealt with health literacy of patients in health settings such as primary care, hospital, rehabilitation and so forth, or in any other way did not match the focus related to occupational health literacy in a corporate context.

The search for relevant literature was conducted in the bibliographical databases Pubmed, Science Direct, and the Directory of Open Access Journals from July 2019 to August 2019. First, abstracts were systematically screened for eligibility in an initial search. In turn, duplicates and abstracts that did not meet the inclusion criteria were removed. Second, the abstracts were assessed to decide on their specific relevance. Full texts were retrieved for those articles fulfilling the inclusion criteria and reviewed in depth by the two researchers. Finally, the data set was compiled based on a consensus approach among the two principal investigators. Only peer-reviewed articles were considered valid for the study. Due to the heterogeneity of the identified references, a thematic analysis was opted for instead of a meta-analysis based on the statistical pooling of data. The data were thematically analyzed, synthesized, and reported in an overview table using Microsoft Excel. Subsequently, thick descriptions were developed for each emerging theme along with a synthesis of the overall results.

## Results

**Figure 1.**
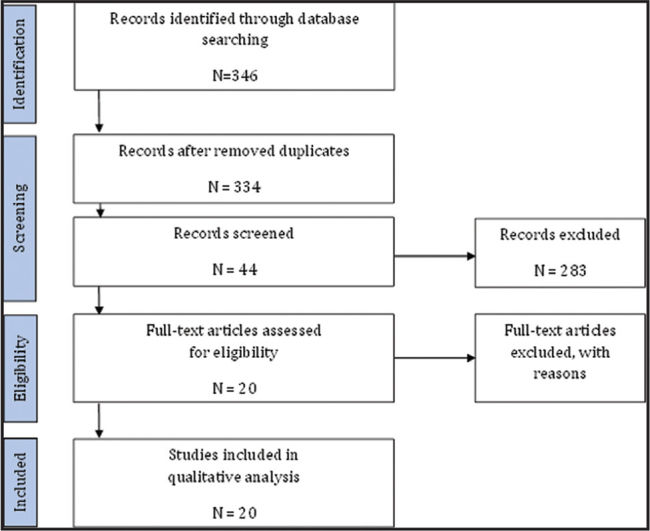
Flowchart regarding the systematic review of health literate companies.

The search strategy resulted in 346 eligible abstracts, as outlined in the PRISMA flowchart in **Figure 1**. After the first scan, duplicates and abstracts not conforming to the inclusion criteria were removed, yielding 334 abstracts. The second review identified 44 abstracts, of which full texts were retrieved. An in-depth assessment of this selection by the research team resulted in 20 articles forming the final data set of the study. The articles were included based on a consensus-approach among the two researchers involved.

Although not all companies could be identified in detail, the profile of the corporations represented in the study was diverse. Companies from Australia, Canada, Germany, and Iran and various professional fields, such as IT (information technology), government, health care, freight transport, and small businesses could be detected.

**Figure 2.**
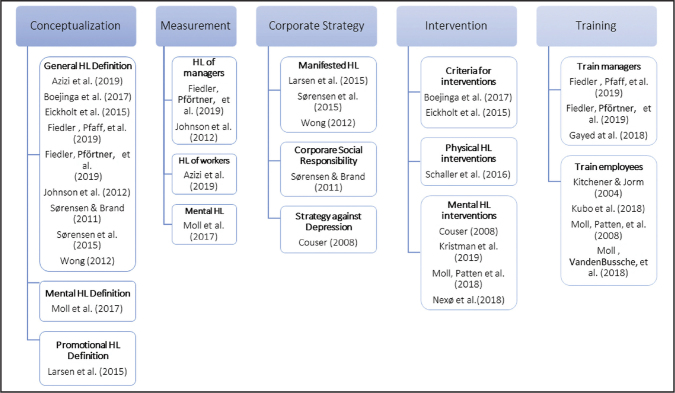
Characteristics of health literacy in a corporate context.

The thematic analysis yielded five clusters with several subgroups describing the characteristics of how health literacy was applied in the corporate context: (1) Conceptualization, (2) Measurement, (3) Corporate Strategy, (4) Intervention, and (5) Training, as illustrated in **Figure 2** and described in detail in the descriptions and the final synthesis below.

### Conceptualization

The literature review revealed that three types of conceptualization of health literacy were applied: (1) general health literacy definitions (Azizi et al., 2019; Boeijinga et al., 2017; Eickholt et al., 2015; Fiedler, Pfaff, et al., 2019; Fiedler, Pförtner, et al., 2019; Johnson et al., 2012; Sørensen & Brand, 2011; Sørensen, Czabanowska, et al., 2015; Wong et al., 2012); (2) a mental health literacy definition (Moll et al., 2017); and (3) a promotional health literacy definition (Larsen et al., 2015). Among the general health literacy definitions, the most widely used included the definitions of the World Health Organization (World Health Organization, 1998) and Sørensen et al. (2012).

### Measurement of Health Literacy

Measurement of health literacy appeared as a separate cluster associated with health literacy interventions at the workplace. The literature review indicated two specific measurement tools presented by Azizi et al. (2019) and Moll et al. (2017). Azizi et al. (2019) developed the Health Literacy Scale for Workers (HELSW), a reliable and valid instrument to assess the level of health literacy in different social groups. With only a few questions and its orientation on occupational health, it makes implementation feasible in work-place settings. It focuses on health literacy and self-efficacy. Moll et al. (2017) described the Mental Health Literacy tool for the Workplace, which is vignette-based and fits the needs of an occupational context, specifically focusing on mental health literacy. Its purpose is to promote early interventions and support for workers with mental health problems.

Furthermore, when measuring the health literacy of managers, business owners, and health professionals, Fiedler, Pförtner et al. (2019) and Johnson et al. (2012) acknowledged essential findings for the integration of health literacy in the occupational context. Fiedler, Pfaff et al. (2019) analyzed both the health-literacy status of managers and the specific criteria for promoting the health literacy of managers. Due to information and work overload, a lack of time and proper priorities, health-wise actions are still low; however, health literacy levels are considerable. Self-responsibility needs more empowerment, and company conditions need to be supportive to let managers show a health-supportive behavior in a health-promoting environment. In turn, Johnson et al. (2012) focused on small business owners, their health literacy, and their potential to act as role models for their social community. Because they found adequate health literacy levels among the business owners, they suggest “using local business owners as disseminators of health-related materials to the communities in which they operate their businesses.”

### Strategy

The literature review indicated that a company's strategic approach to health literacy is important. According to Wong (2012) and Sørensen et al. (2015), integrating health literacy into the strategic thinking of a company is a complex undertaking because many diverse factors need to be taken into consideration, and results are expected in a long-term rather than a short-term perspective. To ensure lasting success, health literacy needs the buy-in of the management and to be seen as a core value beneficial to the corporate determinants of health (Sørensen, Czabanowska, et al., 2015). Also, the study highlighted that strategic workplace policies need to be developed, and health information and services for employees need to be available, reachable, and understandable. This includes the proper addressing of the target group by effective communication and creating incentives to make use of it—what can be done only when the needs of the target group are known and met (Larsen et al., 2015; Sørensen, Czabanowska, et al., 2015; Wong et al., 2012). The managerial level of a company plays a vital role. It provides the resources, by building the infrastructure, hiring trainers, or buying tools, for example, and on the other hand, acts as a role model (Larsen et al., 2015; Sørensen, Czabanowska, et al., 2015; Wong et al., 2012). Moreover, proper health literacy measurement helps in evaluating and improving the progress of the strategy (Sørensen, Czabanowska, et al., 2015). Also, health literacy can be a strategic asset of a company's corporate social responsibility strategy (CSR). Sørensen and Brand (2011) found that companies integrating CSR into their strategy also have useful approaches regarding health and, therefore—effectively—the potential as well to build a health literate workforce. Couser (2008), to mention a concrete example, found that an effective way of preventing depression in the workplace is improving mental health literacy by organizational interventions—manifested in a corresponding strategy.

### Interventions

The focus on workplace interventions constituted a cluster on its own. Eickholt et al. (2015) stressed the importance of encouraging the personal responsibility of employees. They found several criteria that are relevant for the promotion of health literacy in the workplace, such as a supportive environment, enabling informal learning embedded in working processes, and action-oriented concepts that meet the individual needs of various enterprises. Tailoring the intervention to the target group's needs, mindset, and initial health literacy skills were also highlighted (Boeijinga et al., 2017). The importance of physical literacy was emphasized by Schaller et al. (2016), who implemented a behavior-oriented lifestyle intervention and the assignment of a health coach to evaluate a cross-provider workplace-related intervention promoting physical activity and health literacy. Lastly, several studies focused on mental health literacy. Nexø et al. (2018) found in a systematic review some guidelines that include increasing the mental health literacy of all employees and increasing managers' communication and relational competencies to prevent work-related mental health issues. Kristman et al. (2019) developed a practical approach, called the Mental Wellness at Work Project, which was a multi-faceted intervention to improve mental health literacy. Couser (2008) reviewed the evidence supporting factors and interventions for preventing depression in the workplace and highlighted the development of individual resilience, screening of individuals at high risk, improvement of organizational literacy, and an integrated work-place health care system as factors for success. Along this line of thinking, (Moll, Patten, et al., 2018) defined criteria such as organizational support, practical orientation, and contact-based education as success factors for the prevention of mental health problems at work.

### Training

The literature review showed that when integrating health literacy into the occupational setting, training is often considered a specific form of intervention. It was decided, therefore, to separate this cluster from the more classical interventions described in the fourth cluster. Training in the corporate settings involved the management level (Fiedler, Pfaff, et al., 2019; Fiedler, Pförtner, et al., 2019; Gayed et al., 2018) and the employees (Kitchener & Jorm, 2004; Kubo et al., 2018; Moll, VandenBussche et al., 2018). Fiedler, Pfaff, et al., (2019) did not find a beneficial effect on health literacy after their classroom training.

In contrast, Gayed et al. (2018) developed online training to increase mental health literacy among managers as the flexible online courses seemed to suit the demanding workdays of managers better and could be adapted to their learning styles. Training to advance the mental health literacy of employees seemed more prevalent. Notably, the mental health first aid training to improve public mental health literacy including recognition and knowledge of mental health problems, help-seeking behavior, and providing help to someone with a mental health problem was reported to show significant results (Kitchener & Jorm, 2004; Kubo et al., 2018; Moll, Patten, et al., 2018; Moll, VandenBussche et al., 2018). Participants in the studies showed greater confidence in providing help to others, a higher likelihood of advising people to seek professional help, improved concordance with health professionals about treatments, and decreased stigmatizing attitudes (Kitchener & Jorm, 2004; Kubo et al., 2018). Moll, Patten, et al. (2018) recommended that training to increase mental health literacy should be contact-based education with contextually relevant and best practice examples. Furthermore, support from corporate management was required.

## Synthesis

In summary, the systematic literature review indicated that the few initial studies on health literacy in companies generally embrace the concept of health literacy as a stimulus to the development of a corporate culture of health. Notably, the study highlighted that tools exist for measuring health literacy in the context of the workplace, that health literacy of employers is being measured as well as employees, and that it is being used as part of health promotion interventions. It was advised that health literacy strategies be integrated by companies at the management level and throughout the organization and that criteria are developed for tailored interventions within occupational health, physical health, and mental health. Training of staff at all levels is a way of integrating health literacy efforts in practice.

## Discussion

To our knowledge, this study, as the first of its kind, explored how companies apply health literacy to advance health in the workplace. The novel insights disclose that the research in this area is in its infancy. Although the review shows that the concept can be integrated, measured, and applied in the context of health at work, it also highlights the gaps and areas of research that are needed to understand better the role of companies in the advancement of health literacy.

This systematic literature study confirms that health literacy measurement and interventions are useful elements to be applied when improving health in corporate settings. The findings are in line with Conard (2019), who encourages the implementation of health literacy measurement as a quality indicator to show the effectiveness of the interventions by following health metrics and costs over the years. As a proxy indicator, it is evident that health literacy can predict behavior change in self-care, health system engagement, and health outcomes. It is, therefore, strongly encouraged to use health literacy to prove the efficacy of the solutions aimed for (Conard, 2019).

However, it is also paramount that health literacy is researched in a way that is aligned with the World Health Organization and its understanding of a healthy workplace, which is “one in which workers and managers collaborate to use a continual improvement process to protect and promote the health, safety, and wellbeing of all workers and the sustainability of the workplace by considering the following, based on identified needs: (1) health and safety concerns in the physical work environment; (2) health, safety and well-being concerns in the psychosocial work environment including organization of work and workplace culture; (3) personal health resources in the workplace (support and encouragement of healthy lifestyles by the employer); (4) ways of participating in the community to improve the health of workers, their families and members of the community” (World Health Organization, 2011). Advancing health literacy at work entails more than awareness of protection and safety measures. Although the review showed examples of interventions and training provided on physical and mental health, growing a culture of health is much more complex and should be tailored to specific needs within the organization. The study highlights the demand for a much more profound integration and implementation of health knowledge, health behavior, and healthy lifestyle through, for instance, the development of health literate companies.

The managerial level of a company plays a crucial role in the integration of health literacy, and the complexity of the matter demands a framework consisting of high engagement in terms of resources, role-models, and lived values. These findings are in line with the recommendations found for health care organizations (Brach, Dreyer, et al., 2012). Management buy-in is required to ensure a sustainable rise in health literacy among the workforce (Sørensen, Czabanowska, et al., 2015). When done well, health literacy can act as a differentiating factor in highly competitive markets for qualified employees (Sørensen & Brand, 2011).

Looking at today's health challenges, especially on the rising burden of chronic diseases, suggests that a call for sustainable change and health literate societies is needed. The balance between private life and job are on the edge as people spend one-third of their time or more at work. Many chronic diseases are a result of unhealthy lifestyle choices—often in interconnection with their job since fast-paced ways of living have become commonplace. The health literacy of people can be strengthened through lifelong learning, and the workplace setting is beneficial and fit for this purpose. Addressing health literacy as part of a corporate health culture at the workplace gives an excellent opportunity to nurture health literacy, prevent diseases, and promote healthy lifestyles. Even though the criteria for successful health literacy interventions have been defined by Eickholt et al. (2015), the study shows that there is still a lack of practical implementation. The same holds true for the investment in mental health. The investment in mental health literacy as illustrated by this study is an essential way forward to improve health and wellbeing at work (Nexø et al., 2018). To ensure long-lasting success, workplace interventions need to raise overall health literacy as part of a built-in health culture, rather than add-on short-term incentives (Sørensen, Czabanowska, et al., 2015). More investment is warranted for health-literacy-tailored work-place interventions that can lead to the expected outcomes for the workforce.

Although many corporations make positive contributions through their primary activities (McKee & Stuckler, 2018), the growing evidence of the adverse health consequences related to the commercial determinants of health such as marketing, lobbying, corporate social responsibility, and extended supply chains have also come into focus (Kickbusch, 2012). Addressing critical health literacy from a corporate perspective could induce the sustainable development agenda and add to the corporate culture of health as well as the triple bottom line of people, planet, and profit.

Certain limitations of the study need to be acknowledged. First, there is an information asymmetry between studies focusing on mental health literacy at the workplace and general health literacy at the workplace, where the latter is less represented. Second, even though there are several theoretical frameworks for applying health literacy in the occupational setting, only a few have been successfully tested. Fourth, the studies presented are derived mainly from settings in the developed world. Fifth, systematic reviews depend on the validity of the studies included and, therefore, may be affected by possible bias. Sixth, it is likely that many more companies are involved with health literacy activities but have not presented their work in the peer-reviewed literature. Seventh, the language limitations to examine references in only English and German only, may have excluded otherwise suitable references written in other languages.

In conclusion, this systematic review can serve as a starting point for the further development of health literate companies. It is recommended that the corporate sector should continuously engage in the development and integration of health literacy in organizational strategies and priorities. Also, companies can play an active role in the development and evaluation of health literacy interventions tailored to the workforce's need to explore best practices. Realizing that the investment in mental health literacy is essential, it will be beneficial for companies to embrace a broader spectrum of health as recommended by the World Health Organization related to healthy workplaces. More research and practical experience are warranted on how companies and corporations can mobilize and facilitate a health literate workforce.

## What we have Learned

The study revealed five important characteristics of how health literacy is applied in corporate settings: the conceptualization of health literacy, the measurement of health literacy, and the integration of health literacy into strategies, interventions, and training.

With management buy-in, corporations can play an active role in the advancement of health literacy at work.

Due to the emerging phase of this specific field of interest, more research is warranted to elucidate the opportunities and challenges of health literacy as an asset for companies.
